# New classification of maxillary ameloblastic carcinoma based on an evidence-based literature review over the last 60 years

**DOI:** 10.1186/1758-3284-1-31

**Published:** 2009-08-12

**Authors:** Astrid LD Kruse, Roger A Zwahlen, Klaus W Grätz

**Affiliations:** 1 Department of Craniomaxillofacial and Oral Surgery, University of Zurich, Zurich, Switzerland

## Abstract

**Background:**

The ameloblastic carcinoma is a rare malignant odontogenic tumor which rather occurs in the mandible than in the maxilla. Its rarity and in this context somewhat speculative histopathogenesis may account for diagnostic difficulties. Current classifications do not consider benign histopathological features at the primary and malignant features at the metastatic tumour site. Based on an evidence-based literature review, a recommendation for a novel classification is presented.

**Methods:**

An evidence-based literature review over the last 60 years regarding ameloblastic carcinoma of the maxilla was conducted.

**Results:**

An overall of 26 cases were found (mean age: 54.4 (5-83 years); male to female ratio: 2.7 to 1). In 54% the primary diagnosis was ameloblastic carcinoma, 34.6% revealed pulmonary metastases, however, only in one patient cervical lymph node metastasis could be found. Whereas two cases did not reveal malignant histopathology at the primary, they revealed malignant features at their metastatic sites. Nineteen of 26 patients (73,1%) were controlled during a median follow-up time of 54,3 months (6 to 156 months); 6 patients died of disease after a median time of 62,7 months (7 to 156 months) after initial diagnosis.

**Conclusion:**

It is of utmost importance to be aware of that ameloblastomas may be capable to degenerate into a "malignant" disease with recurrence and metastasis. In addition to local long-term control, special attention should be paid to potential pulmonary involvement.

## Introduction

Ameloblastomas, representing 1% of all jaw tumours, are considered to be benign, but locally aggressive odontogenic epithelial neoplasms [1]. The largest review was performed in 1995 by Reichart et al [1] comprising 3677 cases. Amongst others, calcifying epithelial odontogenic tumor, metastatic carcinoma of the jaw and keratocystic odontogenous tumours may come into consideration as differential diagnoses.

The maxillary mandibular ratio of ameloblastoma is 5 to 1, in favour for the mandible. Its most common site of occurrence is the mandibular molar region [1,2]. More than 50% of recurrence appears within the first 5 years after primary surgery[1]. Even though ameloblastomas are well studied and documented, little is known about their malignant features. This is reflected in the fact that whereas more than 3600 cases of ameloblastomas have been described in the literature [3], fewer than 60 cases of ameloblastic carcinoma have been reported, among which two thirds occurred in the mandible.

In regard to malignancy, one must be aware of the difference between malignant ameloblastoma and ameloblastic carcinoma. The latter reveals malignant histopathological features independent of the presence of metastasis [4], whereas malignant ameloblastomas metastasize as well differentiated benign cells [5]. To a high percentage (70-85%) metastases of ameloblastoma occur in the lungs [6,7].

Both aetiology of this rare carcinoma and the question whether this type of carcinoma originates from an ameloblastoma or represents a separate entity are still controversially discussed. However, most of the published data involving ameloblastic carcinoma of the maxilla result from single-case reports; prospective data or data from multi-centre studies are lacking. Treatment guidelines lack of results from long-term follow-up, because most case reports cover a time-span less than 5 years after the primary operation. Moreover, often a long-term follow-up is not possible due to advanced age of patients.

In comparison to ameloblastic carcinomas of the mandible, maxillary ameloblastic carcinomas have not been well studied because of the lack of available data.

Therefore the aim of the here presented evidence based literature review is to collect clinical features and treatment results of maxillary ameloblastic carcinoma over a period of 60 years in order to implement a novel classification for this type of carcinoma.

## Methods

Electronic databases (Medline and Cochrane) were searched using a set of predetermined keywords. The search strategy was initially developed and implemented for PubMed but revised appropriately to suite the other database. A combination of free text terms with Boolean operators and truncation were used. No restriction was placed on the year or language of publication. The search strategy was devised in consultation with a senior librarian.

The citations retrieved from each database were exported into the EndNote bibliometric management software. Duplicates were discarded. The titles and abstracts were screened and the hard copies of all potentially relevant articles were retrieved. Their reference lists were manually searched for any related articles.

## Results

**Figure 1:**
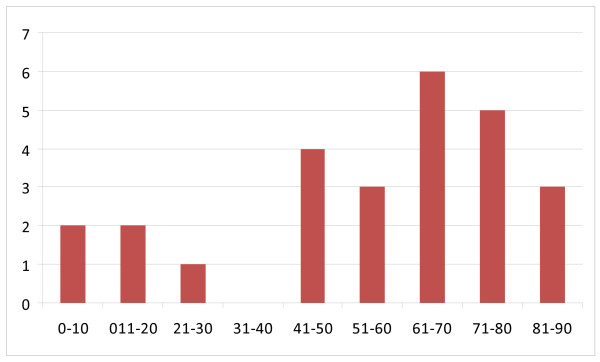
**Age distribution showing the occurrence of maxillary ameloblastic carcinoma**.

**Figure 2:**
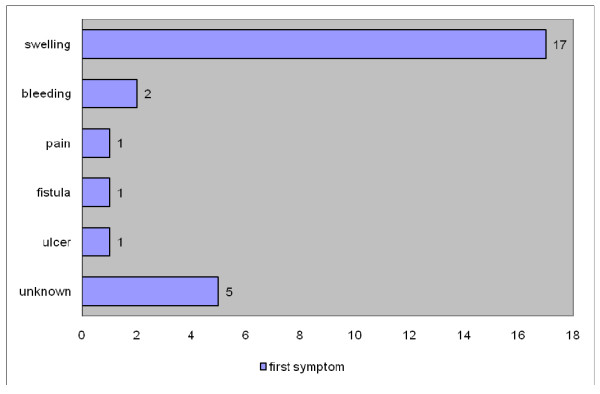
**Distribution of first symptoms**.

**Figure 3:**
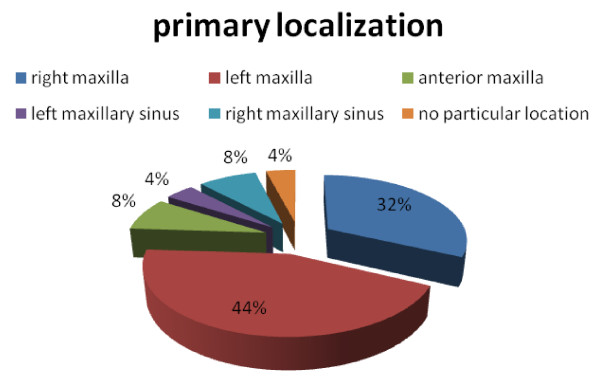
**Distribution of primary tumor localisation**.

**Figure 4:**
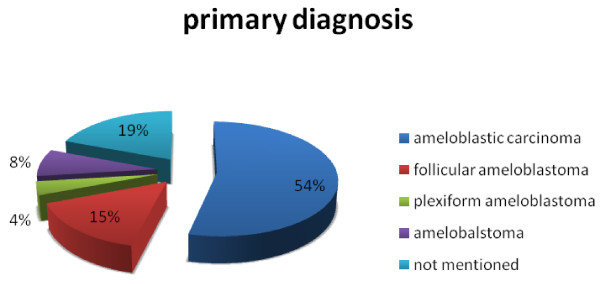
**Histopathological diagnosis of the first specimen**.

**Figure 5:**
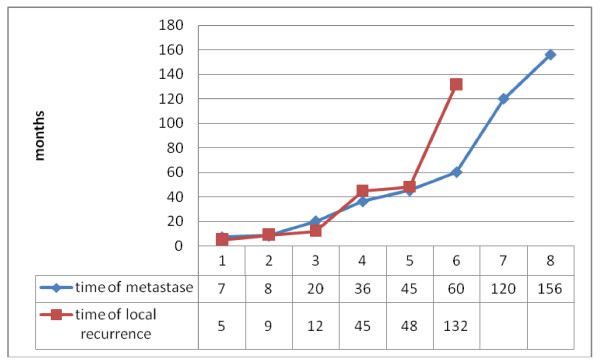
**Time (months) of local recurrence (red) and metastases (blue) appeared**.

During 60 years (1948 - 2008), an overall of 26 cases of maxillary ameloblastic carcinomas have been described (Table 1) [8-28]. In 3 cases only, a description of the autopsy result could be obtained. The mean age was 54.4 years with a marked prevalence in the group from 41 years to 80 years (69.2%) (Fig. 1); female to male ratio was 1 to 2.7. The predominant chief complaint was swelling (Fig. 2). Regarding the tumour localisation, 44% were found in the left maxilla, and 32% in the right side of maxilla (Fig. 3); in 54% the first specimen revealed directly an ameloblastic carcinoma, but in 15% a follicular ameloblastoma was first found (Fig. 4). 26,9% revealed pulmonary metastases and in 23% local recurrence was detected (Fig. 5). 77% of the patients underwent a median follow-up period of 54.3 months (6 - 156 months) while 23% died of disease after a median time of 62,7 (7 months-10 years) months after initial diagnosis (Fig. 5).

**Table 1:** Overview of published cases of ameloblastic carcinoma of the maxilla in 60 years (1948 - 2008) (DF: disease free, nm: not mentioned, DOD: dead of disease)

**Author**	**Year**	**Sex**	**Age**	**treatment**	**metastases**	**Local recurrence**	**Follow-up (months)**	**DOD**
Grimes OF [8]	1948	f	46	Surgical excison	lung		120	NM

Eda S [9]	1972	f	44	Surgical excision	Submand. LN, lung, vertebra	6 times	121	DOD

Krempien B [10]	1979	m	5	Surgical excision	lung		144	DF

Daramola JO [11]	1980	m	22	Surgical excision, radiotherapy, chemotherapy	lung		0	NM

Madiedo G [12]	1981	m	49	Surgical excision, chemotherapy		2 times (12 m, 20 m)	60	DOD

Anderson E [13]	1986	m	73	Surgical excision		48 m	0	NM

Nadimin H [14]	1987	f	15	Surgical excision			0	NM

Corio RL [15]	1987	m	15	Surgical excision			0	NM

Inoue N [16]	1988	f	51	Surgical excision	lung	132 m recurrence	0	

McClatchey KD [17]	1989	f	77	Surgical excision			24	alive

Lee L [18]	1990	m	56	Surgical excision, radiotherapy	lung	5 m	7	DOD

Lolachi CM [19]	1995	f	82	Surgical excision			0	NM

Ingram EA [20]	1996	m	83	Surgical excision			24	alive

Infante-Cossio P [21]	1998	f m m	69 77 64	Surgical excision, radiotherpay Surgical excision Surgical excision, radiotherapy	brain		60 7 36	alive DOD alive

Sastre J [22]	2002	m	40	Surgical excision			24	alive

Avon SL [23]	2003	m	68	Surgical excision	no	No	120	alive

Zwahlen RA [24]	2003	m	44	Surgical excison, Radiotherapy, Chemotherapy (palliative)	Cardial, pulmonal, cerebral	2 times	156	DOD

Dhir K [5]	2003	m	72	Surgical excision			20	alive

Goldenberg D [25]	2004	m	72	Surgical excision, radiotherapy				NM

Philip MP [26]	2005	m m	70 56	Surgical excision, Radiotherapy Surgical excison, radiotherapy			40 8	Alive alive

Yazici N [27]	2006	m	10	Surgical excision			6	alive

Benlyarzid A [28]	2007	m	90	Surgical excision			25	DOD

Ward BB [4]	2007	m	64	Surgical excision			30	alive

## Discussion

Maxillary ameloblastic carcinomas are very rare. Therefore features like metastasis pattern, histopathological factors, and gender predilection - in contrary to amelobalstic carcinomas of the mandible - have only been presented in single case reports. Hence the intention of this study was to collect aspects of clinical appearance and to compare these results with the current classifications of ameloblastic carcinomas. Whereas some authors state no gender predilection in ameloblastomas [2], a preponderance of males was found in the here presented study with a female-male ratio of 1 to 2.7. The first clinical sign in 61.5% of cases was swelling; bleeding, ulceration or fistula was only found in 15.4%. It might be assumed that therefore patients presented already with a progressive form at first sight.

Progressive types of ameloblastic carcinomas may also be associated with the degree of aggressiveness being possibly defined as cortical bone perforation, invasion of soft tissue, recurrences, and metastases. Both pathways haematogenous as well as lymphatic seem to be possible, even though the latter is rare. Among the reviewed cases, 34.6% revealed metastases and 23.1% local recurrences. In only one case neck lymph nodes were involved. In 26.9% pulmonary metastases occurred. This high percentage emphasizes the importance to detect pulmonary metastases either by computertomography or PET-scans as well as the need for long-term follow-up. Besides these screening methods, increasing serum calcium has been considered to be a predictor of metastases [12], even though such an increase might be unspecific due to an osteolysis.

Histopathologically two factors have been discussed as predictors for metastasis and/or aggressive behaviour: granular cell change; an extensive clear cell component [15,29,30]. However, these histopathological features have not been investigated in all case reports and therefore general interpretation is lacking evidence. The difficulty for this seems to be the difference whether the carcinoma is a different entity or whether it has originated from an ameloblastoma.

In the present study (Fig. 4) 50% was determined to be an ameloblastic carcinoma, not excluding, however, the potential development of an undetected ameloblastoma. Especially this problem has not been considered in current classifications (Table 2). Besides the classification of the WHO [31], two other current classifications have been developed (Table 1) so far. A significant disadvantage, however, remains the presupposition that the origin, including the histopathogenesis of ameloblastic carcinoma, is still unknown.

**Table 2:** Classifications of ameloblastic carcinoma by Elzay (1982) and Slootweg & Müller (1984) and the novel classification

**Type**	***Elzay *(1982)**[32]	***Slootweg & Müller *(1984)**[33]	***Kruse et al.(2009)***
**1**	**Arising from an odontogenic cyst**	Primary intraosseous carcinoma ex odontogenic cyst	Malignant ameloblastoma

1a	-	-	Metastase with features of an ameloblastoma (well differentiated)

1b	-	-	Metastase with malignant features (poorly differentiated)

**2**	**Arising from an ameloblastoma**		Ameloblastic carcinoma arising from an ameloblastoma

2a	Well differentiated (malignant ameloblastoma)	Malignant ameloblastoma	Without metastase

2b	Poorly differentiated (ameloblastic carcinoma)	Ameloblastic carcinoma, arising *de novo*, ex ameloblastoma or ex odontogenic cyst	Metastase with features of an ameloblastoma (well differentiated)

2c			Metastase with malignant features (poorly differentiated)

**3**	**Arising *de novo***	Primary intraosseous carcinoma *de novo*	Ameloblastic carcinoma with unknown origin histology

3a	No keratinizing	No keratinizing	Without metastase

3b	Keratinizing	Keratinizing	Metastase with features of an ameloblastoma (well differentiated)

3c			Metastase with malignant features (poorly differentiated)

Therefore, considering its unknown origin and the phenomenon that has been described in the literature [8,10], we recommend a modification of the current classification (Table 2), where a primary ameloblastoma is followed by secondary metastasis with histopathological features of malignancy and without evidence of malignancy in the primary localization.

What treatment armamentarium do we have when dealing with this entity? It is well accepted that maxillary ameloblastomas should be treated as radically as possible due to the spongy maxillary bone architecture. This structure may facilitate the spread of the tumour and may lead to infiltration of adjacent vital structures. In contrast to this, the speed of growth in the mandible is decelerated due to the thick and compact bone structure [3].

Curettage of maxillary ameloblastomas is known to be associated with recurrence in almost 100% of cases [7]. A surgical resection with 10-15 mm margin free of tumour is recommended [34], even though the extent of the resection may be limited related to adjacent pivotal anatomical structures, particularly in the maxilla. Especially in these kinds of localisations it is of utmost importance to be as radical as possible to control recurrence and potential degeneration into ameloblastic carcinoma. Regular follow-up and CT- or MRI controls, in particular in maxillary ameloblastomas, are broadly accepted among clinicians.

Controversy still exists regarding its treatment: some authors have suggested radiotherapy [5,20,21] while others [35,36] doubt its effectiveness. Most of the ameloblastic carcinomas are intraosseous; therefore, the effectiveness of radiation therapy must be considered critically. Philip et al. (2005) [26] suggested to apply adjuvant radiotherapy in patients with positive resection margins, multiple positive lymph nodes, extracapsular spread, perineural invasion, and in patients where salvage surgery would be inefficient. Reports about chemotherapy regimens in ameloblastic carcinoma are rare. In the present evidence based review only 3 patients showing a progressive disease were treated with chemotherapy, and (they both) all of them died.

With respect to aetiology, differentiation from ameloblastomas and an association with recurrence relating to multiple surgical procedures have been discussed [33]. Among all the reviewed cases, 27% presented at first histopathologic diagnosis as a follicular or plexiform ameloblastoma and secondarily as an ameloblastic carcinoma.

The lack of non evidence-based study designs represents a major shortcoming of the here presented evidence based literature review. This prevents to collect information in a standardised manner, however, is in a way to understand due to the rarity of this pathologic entity. Considering the limitations of this study, a remarkable aggressiveness of this pathologic entity could be detected, even though there were considerable differences in respect to the treatment protocols in terms of surgery, postoperative follow-up and period of follow-up among the reviewed studies. The authors suggest that in performing multicenter studies dealing with such rare entities, it would be easier to develop treatment protocols, simply by pooling the cases and experiences of such rare entities.

To provide more information about the biological behaviour of ameloblastic carcinomas, Carinci F et al (2004) [37] described their first genetic portrait. Yet more studies will have to be performed before, apart from surgery, specific adjuvant treatment strategies may be implemented. This novel classification might be a step on the ladder to specify more accurately the original nature of this carcinoma.

## Conclusion

The novel classification considers the unknown origin as well as primary ameloblastomas with metastases and their histopathological features of malignancy without previous evidence of malignancy in the primary localization.

In cases of maxillary ameloblastomas, a radical resection should be performed in order to prevent recurrence and development of malignancy. Patients with maxillary ameloblastomas should undergo a life-long follow-up including regular CT or MRI scans, for early detection of recurrence. For the staging procedure PET scan or chest CT should be performed in order to detect pulmonary metastases. In cases with maxillary ameloblastic carcinoma, a neck dissection should only be performed in the presence of clinically positive lymph nodes.

## Competing interests

The authors declare that they have no competing interests.

## Authors' contributions

AK carried out the analysis of the patients'data, RZ participated in the design of the study and the discussion, KWG participated in the design and coordination of the analysis. All authors read and approved the final manuscript.
